# Comparative Performance of a Routine Immunoturbidimetric Glycated Hemoglobin Assay and High-Pressure Liquid Chromatography Method

**DOI:** 10.7759/cureus.101019

**Published:** 2026-01-07

**Authors:** Faralahy H Rakotonjafiniarivo, Soja M Rakotomalala, Tokinomenjanahary Antsonantenaina, Miora K Ranaivosoa

**Affiliations:** 1 Medical Biochemistry, University of Antananarivo, Antananarivo, MDG; 2 Medical Biology, University of Antananarivo, Antananarivo, MDG

**Keywords:** hba1c, high-performance liquid chromatography, hplc, immunoturbidimetric assay, madagascar, performance

## Abstract

Introduction

Hemoglobin A1c (HbA1c) testing is used for the diagnosis of diabetes and glycemic monitoring. This study compares an immunoturbidimetric assay with high-performance liquid chromatography (HPLC) to assess analytical agreement and determine the clinical suitability of the immunoturbidimetric method for diagnosis versus long-term monitoring in a local setting, in alignment with international standards.

Methods

A comparative cross-sectional study was conducted in a routine laboratory in Madagascar. Samples were tested on the same day using the Bio-Rad D-10 (Bio-Rad Laboratories, Hercules, CA) and Mindray BS-300 (Mindray, Shenzhen, China) automated analyzers. Analytical accuracy was assessed using control materials. The two methods were compared by evaluating the correlation using Pearson's coefficient (r) and linear regression, and the concordance using the Bland-Altman plot and Passing-Bablok regression. The HbA1c results were divided into a total group and subgroups (HbA1c < 6.5%, 6.5-8%, and > 8%).

Results

Intra-assay, inter-assay, and overall precision are satisfactory (coefficients of variation (CV) <3%) for routine use of both techniques. A good overall correlation between the immunoturbidimetric technique and HPLC was found with r = 0.88. The Bland-Altman plot shows a proportional increase in mean bias with HbA1c values and the regression equation $y = 0.8144\,x + 0.3065$.

Conclusion

This study showed good correlation between the two techniques, but a systematic underestimation of HbA1c levels by the immunoturbidimetry assay was observed, especially for high values.

## Introduction

Glycated hemoglobin (HbA1c) testing plays a central role in the diagnosis and monitoring of diabetes and in predicting the risk of complications, provided that standardized methods are used and targets are individualized according to the patient's profile [1,2]. The National Glycohemoglobin Standardization Program (NGSP) and the International Federation of Clinical Chemistry (IFCC) have designated high-performance liquid chromatography (HPLC) as the reference method for measuring HbA1c [3,4]. The turbidimetric method is often preferred for HbA1c testing because it is inexpensive and easy to use [5]. In a resource-limited country such as Madagascar, comparing the local performance of the turbidimetric method with the HPLC method ensures the quality of diabetes diagnosis, monitoring of diabetics, and management of complications, as well as guaranteeing alignment with international standards.

This study aims to evaluate the concordance, correlation, and bias between the HPLC method on D-10 (Bio-Rad Laboratories, Hercules, CA) and the immunoturbidimetric method on the BS-300 automated analyzer (Mindray, Shenzhen, China) for HbA1c testing.

## Materials and methods

Type and location of the study

This is a comparative, cross-sectional, analytical study conducted at the biochemistry laboratory of a tertiary care hospital in Antananarivo, Madagascar, in September 2025.

Sample collection

The blood samples used came from residual specimens from patients who had undergone HbA1c testing in a private health center laboratory using the HPLC reference technique. A total of 63 blood samples were studied. All samples were collected in tubes containing ethylenediaminetetraacetic acid (EDTA). The samples were studied on the same day to ensure the analytical stability of the parameters measured. The data were anonymized, and this study was conducted in accordance with the ethical principles of the Declaration of Helsinki and received approval from the Ethics Committee of the Malagasy Society of Clinical Biology (Société Malgache de Biologie Clinique (SOMABIO)).

Evaluation of analytical accuracy

The results are expressed as percentages (%) with the following conversion: $\text{HbA1c (mmol/mol)} = (\text{HbA1c (\%)} - 2.15) \times 10.929$ [6].

The control materials used for each method were as follows: for the HPLC method, Diabetes Control 1 (5.3 ± 0.13%; Lot 85891) and Diabetes Control 2 (11.03 ± 0.43%; Lot 85892), and for the immunoturbidimetric method, HbA1c-T Control N-I (6.1 ± 0.9; Lot 39102) and HbA1c-T Control N-II (11.0 ± 1.1%; Lot 39103).

HbA1c assay methods

HbA1c levels were measured on the same day using both methods. For the HPLC method, the D-10 automated system (Bio-Rad) was used for the reference technique. This technique is based on the separation of different hemoglobin fractions according to their electrical charges [7]. HbA1c, formed by non-enzymatic glycation of the N-terminal valine of the β chain of hemoglobin A, has a slightly different charge than HbA0. The hemolyzed sample is injected into a cation exchange column and then eluted using a buffer gradient system. The fractions are detected by absorbance at 415 nm and represented as a chromatogram. The D-10 Manager software identifies the peaks corresponding to HbA1c and HbA0, then calculates the percentage of HbA1c relative to total hemoglobin. The analysis time for each sample is approximately five minutes. The D-10 HPLC system is fully traceable to both NGSP and IFCC reference standards. The instrument was calibrated daily using low and high HbA1c control materials provided by the manufacturer, ensuring measurement accuracy across the analytical range. Internal quality controls were run with each batch of samples to monitor precision and stability. For the immunoturbidimetric method, HbA1c was measured using an Automate BS-300 (Mindray). The HbA1c-Turbidimetric reagent from Cromatest (LINEAR Chemicals, SLU, Barcelona, Spain) is based on latex turbidimetry [8]. The latex particles adsorb total hemoglobin and HbA1c (non-specific). The anti-HbA1c monoclonal antibody binds to the latex particles coated with HbA1c. The anti-mouse IgG polyclonal antibody causes agglutination. The resulting turbidity is proportional to the hemoglobin concentration, and the complex is measured at a wavelength of 650 nm. The analysis time for each sample is approximately 10 minutes.

For both measurement methods, HbA1c results are divided into three groups based on target thresholds and mortality risks [2,9]: Group 1: HbA1c < 6.5% (< 48 mmol/mol); Group 2: HbA1c: 6.5-8.0% (48-64 mmol/mol); Group 3: HbA1c > 8.0% (> 64 mmol/mol).

Statistical analyses

Intra-assay, inter-assay, and overall precision were evaluated for both techniques using high- and low-level HbA1c quality controls. Analytical precision was evaluated using the 5x5 method, which consists of performing five consecutive measurements of the same control sample per day for five consecutive days, in accordance with the Clinical and Laboratory Standards Institute (CLSI) EP05 A3 recommendations [10].

Comparison of methods

The correlation between the two assay methods was evaluated by linear regression using Pearson’s correlation coefficient (r), which assesses the strength and direction of the association between the measured values. The relationship between the two assay methods in all groups was also studied using the Passing-Bablok regression analysis [11], which provides regression lines and allows detection of the constant difference (intercept (a)) and proportional difference (slope (b)) with a 95% confidence interval. The concordance between the turbidimetric method and the HPLC method was evaluated using the Bland-Altman plot [12], which allows visualization of the mean bias and the limits of agreement between measurements.

## Results

Accuracy

Table 1 shows the coefficients of variation (CV) according to control levels for both techniques. For the HPLC method, the results of the series of measurements for HbA1c show that intra-series accuracy varies from 0.48 to 1.08% for the high control level and from 0.00 to 1.65% for the low level. The inter-series precision for the two control levels was 0.60 and 1.62, with overall CVs of 1.02% and 1.88% for the high and low levels, respectively. For the immunoturbidimetric method, intra-assay precision ranged from 0.52 to 1.00% for the high level control and from 0.65 to 2.35% for the low level control. The inter-assay precisions are 2.53% and 2.04% for the two control levels, and the overall CVs are 2.65% and 2.41%, respectively.

**Table 1:** Coefficients of variation (CV) according to control levels for both HPLC and immunoturbidimetric methods. Intra- and inter-assay precision values are presented for high and low control levels. Control materials: HPLC – Diabetes Control 2 (11.03 ± 0.43%; Lot 85892) and Diabetes Control 1 (5.3 ± 0.13%; Lot 85891); Immunoturbidimetry – HbA1c-T Control N-II (11.0 ± 1.1%; Lot 39103) and HbA1c-T Control N-I (6.1 ± 0.9%; Lot 39102). HPLC: high-performance liquid chromatography; HbA1c: glycated hemoglobin.

Methods	Control levels	Intra-assay CV (%)	Inter-assay CV (%)	Overall CV (%)
HPLC	High	0.48 – 1.08	0.60	1.02
	Low	0.00 – 1.65	1.62	1.88
Immunoturbidimetry	High	0.52 – 1.00	2.53	2.65
	Low	0.68 – 2.35	2.04	2.41

Correlation

Overall, the Pearson correlation coefficient (r) is 0.88 with r^2^ = 0.774. For the total group, the linear relationship between the two methods was evaluated by Passing-Bablok regression with an equation $\,y = 0.8144\,x + 0.3065\,$ with a 95% CI: −1.0000 to 1.0118 for the intercept (a) and 0.7059 to 1.0000 for the slope (b) as reported in Table 2.

**Table 2:** Passing-Bablok regression analysis and Spearman's rank correlation study of HbA1c results obtained by HPLC and immunoturbidimetric methods. The linear relationship between the two methods was evaluated using Passing-Bablok regression [11] and correlation analysis using Spearman's rank correlation. Intercept (a) and slope (b) with 95% confidence intervals are presented for the total group and three subgroups. P-values < 0.05 were considered statistically significant. HPLC: high-performance liquid chromatography; HbA1c: glycated hemoglobin.

Group	Intercept (a) (95% CI)	Slope (b) (95% CI)	Correlation coefficient (95% CI)	p
Total	0.3065 (-1.0000 – 1.0118)	0.8144 (0.7059 – 1.0000)	0.88 (0.80 – 0.92)	<0.001
Group 1 (HbA1c < 6.5%)	-7.05 (-24.3000 – -4.3876)	3.25 (1.6250 – 5.0001)	0.65 (0.34 – 0.83)	0.004
Group 2 (HbA1c 6.5 – 8.0%)	-6.6667 (-21.5500 – -0.6000)	1.8333 (1.0000 – 4.0000)	0.48 (0.07 – 0.74)	0.02
Group 3 (HbA1c > 8.0%)	1.5848 (-0.8111 – 3.3000)	0.6413 (0.4444 – 0.8889)	0.94 (0.82 – 0.98)	0.0001

Concordance

**Figure 1:**
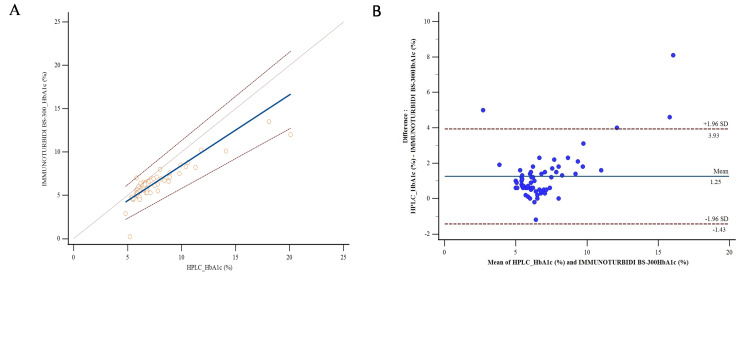
Method comparison in the total group. (A) Passing-Bablok regression graph showing the linear relationship between HPLC and immunoturbidimetric methods [11]. (B) Bland-Altman plot illustrating the agreement between the two methods, with mean differences and limits of agreement [12]. HPLC: high-performance liquid chromatography; HbA1c: glycated hemoglobin.

**Figure 2:**
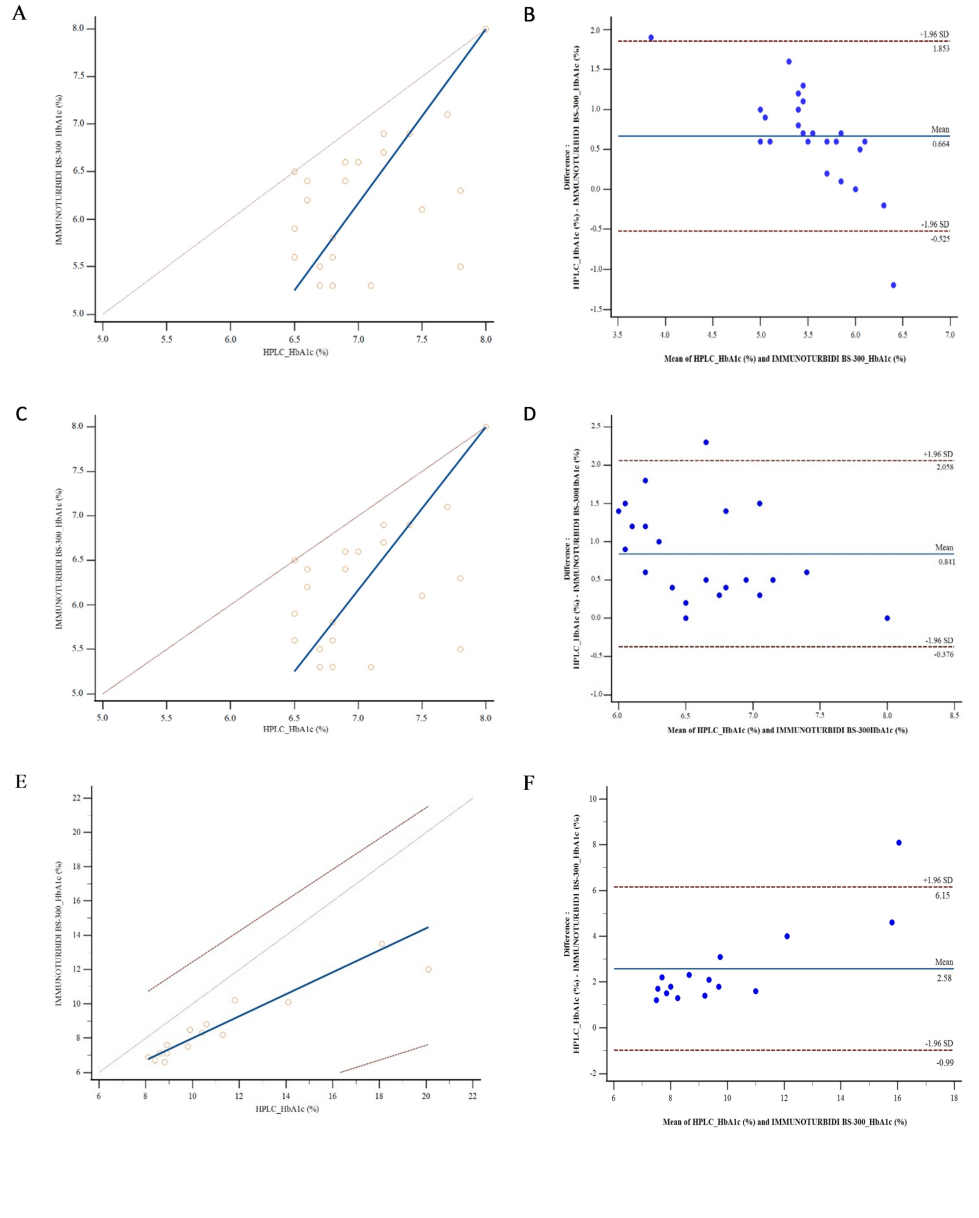
Method comparison in three subgroups. (A, C, E) Passing-Bablok regression graphs of HPLC and immunoturbidimetric methods for Group 1 (HbA1c <6.5%), Group 2 (HbA1c 6.5–8.0%), and Group 3 (HbA1c >8.0%), respectively. (B, D, F) Bland-Altman plots for the corresponding groups showing mean differences and limits of agreement [12]. HPLC: high-performance liquid chromatography; HbA1c: glycated hemoglobin.

Table 3 shows the means and standard deviations for the total group and the three subgroups. In the comparative analysis of HbA1c measurement methods, quantitative data were summarized using the mean and standard deviation, as these indicators respectively reflect the central tendency and variability of the measurements produced by each method. Figures 1, 2 show the Bland-Altman plot between the two methods for the total group and three subgroups. This graphical method evaluates the agreement between the techniques by assessing the mean difference and the limits of agreement. It also makes it possible to detect outliers or trends that might indicate methodological discrepancies. The mean biases are +0.664%, +0.841%, and +2.588% for Group 1 (HbA1c <6.5%), Group 2 (HbA1c 6.5-8.0%), and Group 3 (HbA1c >8.0%), respectively.

**Table 3:** Comparison of mean and median HbA1c results in total and in three subgroups obtained by HPLC and immunoturbidimetric methods. Quantitative data are summarized using the mean and SD to reflect the central tendency and variability of measurements. Statistical significance was assessed for group comparisons (p < 0.05). HPLC: high-performance liquid chromatography; HbA1c: glycated hemoglobin.

Group	No.	Statistical analysis	HbA1c (%) by HPLC method	HbA1c (%) by immunoturbidimetric method
Total	63	Mean	7.53	6.28
		SD	2.75	1.93
		Median	6.7	5.9
		p	<0.001	
Group 1 (HbA1c <6.5%)	26	Mean	5.83	5.20
		SD	0.35	0.77
		Median	5.9	5.2
		p	<0.001	
Group 2 (HbA1c 6.5–8%)	22	Mean	7.04	6.20
		SD	0.47	0.69
		Median	6.9	6.25
		p	<0.001	
Group 3 (HbA1c > 8%)	15	Mean	11.19	8.61
		SD	3.59	2.03
		Median	9.9	8.2
		p	<0.001	

## Discussion

This study was conducted with the aim of comparing the results of HbA1c measurement using the HPLC reference technique and the immunoturbidimetric technique routinely used in a laboratory at a tertiary care hospital in the Malagasy capital.

According to international recommendations (NGSP and CLSI), the acceptable CV for HbA1c measurement is < 1.5% for intra-assay, ≤ 2% for inter-assay, and < 3% for overall CV [10,13,14]. Thus, the majority of measurements performed in both laboratories using both methods (turbidimetric and HPLC) comply with international standards, with good daily repeatability and excellent inter-day stability. The two methods used for HbA1c measurement remain sufficiently reliable for daily clinical use in the diagnosis and exclusion of diabetes.

All HbA1c test results were analyzed overall (in the total group) and according to the three subgroups corresponding to the non-diabetic threshold (Group 1: HbA1c <6.5%), the threshold for diabetics with good glycemic control (Group 2: HbA1c 6.5-8.0%), and the threshold for diabetics with hyperglycemia (Group 3: HbA1c >8.0%). For all subgroups, the HbA1c averages obtained using the turbidimetric method were lower than the averages obtained using the HPLC method. This is consistent with the results of previous studies [15-17]. This trend has also been reported in diabetic patients with chronic kidney disease [18]. The correlation between the two measurement methods was strong, with a Pearson correlation coefficient of r = 0.88, corresponding to a coefficient of determination r² ≈ 0.77, indicating that 77% of the variation in the measured values was explained by the linear relationship between the methods. The regression equation indicates a small constant bias (intercept ≈ 0.3065) and a significant proportional bias (slope < 1), suggesting that in this study, the immunoturbidimetric technique is generally correlated with the HPLC technique, but there is a slight underestimation of high values.

In the total group, the Bland-Altman plot shows a positive mean bias of +1.25%, indicating that the immunoturbidimetric method underestimates HbA1c compared to HPLC. The proportional increase in mean bias with HbA1c values according to the three subgroups (HbA1c <6.5%, HbA1c 6.5-8.0%, and HbA1c >8.0%) confirms that the immunoturbidimetric method increasingly underestimates high values compared to HPLC. The HbA1c ranges between 6.5-8.0% and above 8.0%, which are more markedly underestimated by the immunoturbidimetric method, correspond precisely to the values used for diabetes monitoring and for assessing glycemic control in patients with diabetes. This underestimation, therefore, represents a limitation of the immunoturbidimetric technique, in a local context, for the follow-up of diabetic patients. Klingenberg et al. also observed a generally good correlation, showing that immunoturbidimetry follows the general trend of the values provided by HPLC, but the immunological method showed a systematic bias, with a slight underestimation or overestimation depending on HbA1c levels [19]. Clinically, an increase in HbA1c correlates proportionally with an increase in microvascular and macrovascular complications [20,21]. On the other hand, the immunoturbidimetric technique is more prone to analytical interference, particularly the presence of hemoglobin variants [22,23], which requires caution when interpreting the results.

Limitations of the study

Although no hemoglobinopathy was mentioned in the patients' medical records, the hemoglobin profile and its quantification were not determined, which made it impossible to take into account the possible interference of hemoglobin variants on the HbA1c assay. Hemoglobin variants, particularly elevated fetal hemoglobin, can cause method-dependent underestimation of HbA1c, potentially leading to misinterpretation of glycemic control, while common variants such as HbS, HbC, HbD, and HbE may have minimal impact depending on the analytical system used [22]. Also, HbA1c levels are often higher in certain racial and ethnic groups (Black, Asian, and Latino populations) compared with White individuals, even at equivalent glycemic levels, which may influence both the diagnosis and management of diabetes. From another perspective, these differences highlight the need to consider biological and genetic factors when interpreting HbA1c results [24]. Several non‑glycemic clinical conditions and comorbidities are recognized to influence HbA1c measurements independently of actual glycemia. These include conditions that alter erythrocyte lifespan (e.g., anemia and increased erythropoiesis), pregnancy, chronic liver disease, endocrine disorders like thyroid disease, and certain infectious or chronic inflammatory states, among others, which can result in falsely elevated or decreased HbA1c values depending on the analytical method used. Such confounders should be considered in the interpretation of HbA1c results and when comparing analytical methods [25].

## Conclusions

In this study of a Malagasy population, the immunoturbidimetric technique showed good correlation with HPLC for the diagnosis or exclusion of diabetes, but it tends to underestimate HbA1c at higher glycemic levels, limiting its use for monitoring diabetic patients. Therefore, confirmation by HPLC or another separative method is recommended in clinical situations requiring maximum accuracy.
